# Influence of the Tumor Microenvironment on Cancer Cells Metabolic Reprogramming

**DOI:** 10.3389/fonc.2018.00117

**Published:** 2018-04-19

**Authors:** Victoire Gouirand, Fabienne Guillaumond, Sophie Vasseur

**Affiliations:** ^1^Centre de Recherche en Cancérologie de Marseille (CRCM), UMR 1068, Institut National de la Santé et de la Recherche Médicale, Marseille, France; ^2^Institut Paoli-Calmettes (IPC), Marseille, France; ^3^Unité Mixte de Recherche (UMR 7258), Centre National de la Recherche Scientifique (CNRS), Marseille, France; ^4^Université Aix-Marseille U105, Marseille, France

**Keywords:** microenvironment, metabolism, tumor, metastasis, protein scavenging, fibroblasts

## Abstract

As with castles, tumor cells are fortified by surrounding non-malignant cells, such as cancer-associated fibroblasts, immune cells, but also nerve fibers and extracellular matrix. In most cancers, this fortification creates a considerable solid pressure which limits oxygen and nutrient delivery to the tumor cells and causes a hypoxic and nutritional stress. Consequently, tumor cells have to adapt their metabolism to survive and proliferate in this harsh microenvironment. To satisfy their need in energy and biomass, tumor cells develop new capacities to benefit from metabolites of the microenvironment, either by their uptake through the macropinocytosis process or through metabolite transporters, or by a cross-talk with stromal cells and capture of extracellular vesicles that are released by the neighboring cells. However, the microenvironments of primary tumor and metastatic niches differ tremendously in their cellular/acellular components and available nutrients. Therefore, cancer cells must develop a metabolic flexibility conferring on them the ability to satisfy their biomass and energetic demands at both primary and metastasis sites. In this review, we propose a brief overview of how proliferating cancer cells take advantage of their surrounding microenvironment to satisfy their high metabolic demand at both primary and metastasis sites.

## Introduction

Cellular heterogeneity of solid tumors strongly impacts tumor progression. This abundant hetero-cellularity drives the nature and abundance of the components of the extracellular matrix (ECM) and, for some cancers, makes tumor cells the minor cell type in terms of cellular amount. It appears that tumor cells take advantage of this dense microenvironment and are engaged in a complex dialog with their surrounding cells. A multitude of studies have emerged to dissect the inflammatory, metabolic, or oncogenic nature of the dialog between different cell types in tumors and have improved our knowledge of the various communication modes between cells; physical interactions, secreted molecules, extracellular vesicles (EVs), etc. In this review, we highlight how the stroma [mainly cancer-associated fibroblasts (CAFs)]-tumor cell metabolic axis increases the metabolic performance of tumor cells in addition to the cell’s autonomous metabolic pathways. We also discuss how tumor cells recycle some metabolites, considered until now as “metabolic wastes,” to support their biosynthetic and bioenergetic needs. Finally, we point out the metabolic plasticity that metastatic tumor cells acquire to adapt to the microenvironments of both the primary and metastasis sites.

## Metabolic Communication Between Tumor Cells and Their Neighboring Cells

**Figure 1:**
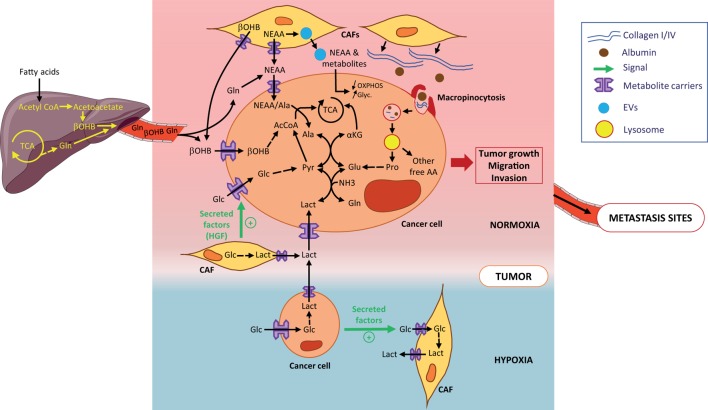
Metabolic symbiosis and recycling in primary tumors. Tumor cells obtain metabolites and AA either from the circulation or from the local microenvironment. Metabolites, such as β-OHB or Gln are released into the circulation by the liver and reach the normoxic tumor (pink zone). At the primary tumor site, AA and other metabolites are locally released by cancer-associated fibroblats (CAFs) (yellow cells). Tumor cells (light brown cells) directly take up AA and metabolites through metabolite carriers or indirectly by (1) uptake of metabolites-loaded EVs from CAFs and (2) macropinocytosis of extracellular matrix such as collagen or scavenging of macromolecules such as albumin. Macropinosomes are internalized and fused with lysosomes where collagen and albumin are degraded into free proline and free AA, all released in the cytosol. Lactate secreted by hypoxic tumor cells (localized in blue zone) is taken up by normoxic tumor cells through the metabolic symbiosis. The most part of AA and metabolites provided to tumor cells are used to contribute to the TCA. All are shown to contribute to tumor growth and metastatic potential of tumor cells. Abbreviations: AAs, amino acids; NEAAs, non-essential amino acids; Ala, alanine; Glc, glucose; Pyr, pyruvate; Lact, lactate; αKG, α-ketoglutarate; Glu, glutamate; Gln, glutamine; β-OHB, β-hydroxybutyrate; EVs, extracellular vesicles; TCA, tricarboxylic cycle; OXPHOS, oxidative phosphorylation; Glyc, glycolysis.

Cancer-associated metabolic remodeling is not restricted to malignant cells but is also found in tumor-surrounding, non-transformed stromal cells. This stromal metabolic reprogramming is dictated by the tumor cells and, as a feedback loop, microenvironmental cells drive metabolic changes in tumor cells and/or provide metabolic resources required for tumor growth. CAFs are the most prominent cell type in the tumor microenvironment (TME) (1, 2) and have emerged as key components of the stromal-epithelial metabolic coupling. In pancreatic cancer cells, Sherman et al. have shown through a transcriptomic approach that soluble cues from patient-derived CAFs induce deep metabolic alterations that are similar to those driven by K-RAS. The metabolic pathways found the most enriched in stroma-activated tumor cells are associated with steroid and unsaturated fatty acid biosynthesis, and also with glycolysis and gluconeogenesis. Moreover, stromal cues increase flux through glycolysis and pentose phosphate pathway (PPP) and enhance tricarboxylic acid (TCA) cycle intermediates. Hence, the stroma induces genomic and metabolic responses that strengthen pancreatic tumor progression (3). A recent *in situ* study analyzing single cell enzymatic activities in intact tumor tissues showed that CAFs have a higher glycolytic activity than the different subtypes of breast tumor cells (4). CAFs metabolize glucose through anaerobic glycolysis and export lactate which is then taken up by oxidative cancer cells to increase their tumorigenic potential (5), a phenomenon coined the “reverse Warburg effect” which is also reported in other cancers (6) (Figure 1). The CAFs’s-enhanced glycolysis is induced by cancer cells in response to hypoxic or oxidative stress injuries (7, 8) and is not a general adaptive metabolic feature of CAFs. Indeed, even if pancreatic-CAFs are prone to glycolysis (9), the lactate is secreted at a lower rate than alanine, a non-essential amino acid (NEAA) (10). Secretion of alanine by CAFs results from autophagy-induced protein breakdown in these cells and, once taken up by cancer cells, it has an unusual metabolic rate. Instead of contributing to protein synthesis, alanine is converted into pyruvate in the mitochondria to provide energy and lipids essential to pancreatic-ductal adenocarcinoma (PDAC)-cell survival and growth. In breast tumors, CAFs release kynurenine, a tumor-promoting metabolite arising from tryptophan breakdown, whose synthesis is increased in response to tumor-derived lipid mediators (prostaglandin E2) (11). In these tumors, transcriptional profiles of caveolin-1-deficient fibroblasts reveal an over-expression of ketogenic genes promoting ketone bodies (KB) production. Hence, CAFs contain a pool of KB that can be used as an energy source by oxidative breast tumor cells, and that can promote breast tumor growth (Figure 1) (12). CAFs also mediate tumor cells’ metabolic reprogramming in a paracrine manner through diffusible or EV molecules. CAF-derived EVs promote a metabolic switch from mitochondrial oxidative phosphorylation (OXPHOS) to aerobic glycolysis to satisfy prostate and pancreatic tumor cell needs in ATP (Figure 1). Hence, EVs increase reductive glutamine’s contribution to lipogenic acetyl-CoA cycle and the use of extracellular acetate as an additional carbon source for fatty acid synthesis. These metabolic changes are induced by intra-EV metabolites and miRNA-targeting OXPHOS genes (13). CAF-secreted cytokines, such as hepatocyte growth factor, have also been reported to favor glucose uptake in human breast tumor cells by increasing glucose transporter 1 levels (14) (Figure 1).

In tumors, nutrient-deprived conditions strengthen the stromal-epithelial metabolic shares. Under glutamine restriction, patient-derived CAFs survive and undergo a metabolic reprogramming leading to an increase in glutamine synthesis. Enhanced glucose entry into the TCA cycle, along with amino acid (AA) and lactate intake, contribute to the carbon supply for glutamine synthesis, while the nitrogen donors are branched-chain amino acids, NEAA, and ammonia. Once secreted and taken up by ovarian cancer cells, glutamine rescues tumor growth by increasing expression of genes involved in cell cycle, fatty acid, and nucleotide synthesis (15). Under AA and pyruvate starvation, CAFs enhance the packaging of cargo metabolites into EVs to rescue prostate and pancreatic tumor cell survival (13). As metabolites present in CAF-EVs are in their active form, their impact on pancreatic cell proliferation is instantaneous and strong (e.g., exosomal metabolites contribute to one-third of the TCA cycle flux), but short term (16). Interestingly, the EV-metabolite supply is K-RAS-independent (13) suggesting that CAF-mediated metabolic remodeling may be a common trait of cancers.

In addition to metabolic dialog with stromal cells, tumors cells exchange diverse metabolites between one another, a phenomenon initially referred to as metabolic symbiosis. One of the most described processes of metabolic symbiosis relies on the potential of malignant cells to increase their glucose consumption and produce large amounts of lactate through aerobic glycolysis (the Warburg effect). In hypoxic tumors, such as PDAC, large amounts of lactate secreted by highly glycolytic hypoxic cells are taken up by normoxic neighboring tumor cells and promote their proliferation. Lactate thereby contributes to the metabolic symbiosis between both hypoxic and normoxic cellular compartments of the tumor (17) (Figure 1). In non-small-cell lung cancers, Faubert et al. showed that tumor cells also metabolize lactate, proving lactate’s contribution as a fuel of the TCA cycle exceeds that of glucose *in vivo* (18). Treatment of pancreatic neuroendocrine, breast and renal cancers with angiogenic inhibitors induces regionalization of these tumors into hypoxic and normoxic zones and force normoxic cells, in close proximity to hypoxic ones, to operate a metabolic symbiotic shift toward the use of lactate diffusing from hypoxic regions. Interestingly, lactate catabolism by normoxic tumor cells is branched to the glutamine metabolism through lactate-derived pyruvate transamination. This reaction allows the production of alanine and α-ketoglutarate, thereby fueling the TCA cycle (Figure 1). This symbiotic metabolism is dependent on mTOR signaling as treatments of tumors with a combination of angiogenic inhibitors and the mTOR inhibitor rapamycin suppresses lactate catabolism by normoxic cells. Normoxic cells switch their metabolism back to a more glycolytic phenotype to the detriment of hypoxic cells which are then devoid of bio-available glucose (19).

Interestingly, lactate not only participates in metabolic processes but also contributes to oncogenic signaling pathways. This function is not restricted to lactate as many by-products of metabolic pathways act as signaling molecules (20). This notion of the dual function of metabolites, being involved in metabolic and oncogenic signaling, strengthens the tight association between tumor metabolism, cell cycle dysregulation, and aberrant cell proliferation. Interestingly, these processes are directed by the circadian clock which governs biological rhythms for tissue homeostasis (21, 22). Hence, an integrated and dynamic view of how metabolic pathways and cell cycle machinery interact with the circadian clock in the context of tumor progression deserves to be better explored, and would help to develop efficient metabolic therapeutic strategies as circadian timing of drug administration impacts both the efficacy and the toxicities of most pharmacotherapies (23).

## Metabolic Recycling

Increased hetero-cellularity of the TME is associated with substantial ECM deposition. This densification of the ECM during tumor growth is associated with changes in stiffness, elasticity, and mechanical properties of the microenvironment but also increases the richness of macromolecules surrounding the tumor cells. Hence, the TME becomes a nutrient supply center for the tumor cells as it is composed of abundant macromolecules, such as collagen, hyaluronan, fibronectin, albumin, lipids, etc. To take advantage of this enriched microenvironment and optimize the use of the macromolecules, tumor cells use macropinocytosis, a non-selective endocytic process, to take up extracellular components and internalize them into vesicles. Products derived from successive degradation of the vesicles’ contents are released in the tumor cells’ cytosol as ready-to-use nutrients. Macropinocytosis occurs in several types of cancers and is largely described as a central process of acquisition of nutrients by PDAC cells. *In vivo*, inhibition of macropinocytosis impedes the growth of subcutaneous xenografted pancreatic tumors (24). Vander Heiden’s lab also demonstrated that PDAC satisfies its avidity for macromolecules such as albumin using macropinocytosis and locally increases its breakdown to derive pools of AAs (25). In this context, K-RAS appears to be a main oncogenic driver of this process. *In vitro*, K-RAS mutant PDAC cells compensate for a lack in AAs in culture medium and maintain a high proliferative rate due to their capacity to recover the needed AAs pools from scavenged and catabolized albumin (26). Interestingly, the ability of spontaneous PDAC to take up macromolecules is not only restricted to albumin but also includes fibronectin. Recently, we also showed that PDAC cells, when deprived of nutrients, scavenge environmental collagen which appears essential for their survival especially under glucose deprivation, the condition in which macropinocytosis is activated. Subsequent digestion of collagen supplies PDAC cells with pools of proline, a main component of collagen molecules. Proline catabolism by PRODH1 enriches the cells with TCA intermediates, especially under nutrient deprivation, and appears to be a promoting metabolic pathway of PDAC growth (Figure 1) (27).

Proliferative tumor cells can also recycle metabolites found in the circulation, such as acetate and KB. Both have been described to be carbon sources to supply the tumor with energy and biomass. Breast cancer cell lines consume large amount of acetate when subjected to metabolic stress and hypoxia to synthesize fatty acids and supply the membrane with phospholipids. Acyl-CoA short chain synthetase 2 promotes the acetate uptake and produces the acetate-derived acetyl-CoA pool needed for fatty acids synthesis (28). KB represented by acetone, acetoacetate, and β-hydroxybutyrate (β-OHB) constitute another family of metabolites that are usable by tumor cells. In physiological conditions, KB are produced in liver, released into the circulation and reach tissues/organs to supply cells in biomass and energy especially in low glucose condition. KB catabolism (ketolysis) consists in oxidation of β-OHB, the most abundant KB in the circulation, to form acetoacetate which is converted by 3-oxoacid CoA-transferase 1 (OXCT1) to produce successively acetoacetyl-CoA and 2 molecules of acetyl-CoA. The latter directly fuels the TCA cycle and supplies cells with ATP. In human hepatocellular carcinoma (HCC), tumor cells overexpress OXCT1 when nutrient-deprived, suggesting that these cells, contrary to the normal hepatocytes counter-part which synthesize and produce KB, catabolize β-OHB when they are nutrient stressed. Indeed, OXCT1 favors β-OHB uptake by HCC cells which use β-OHB to fuel their TCA cycle and produce ATP to promote their proliferation (Figure 1). Moreover, OXCT1 expression in HCC cells is dependent on the mTORC2-AKT-SP1 signaling axis and induction of ketolysis by OXCT1, by supplying cells with ATP, suppresses AMPK activation upon nutrient starvation, avoiding the deleterious excessive autophagy and promoting HCC cell survival and proliferation (29). In melanoma and leukemia driven by the BRAF-V600E mutation, acetoacetate levels are increased. In tumor cells, acetoacetate enhances BRAF-V600E binding to MEK1 and thereby activates the MEK–ERK signaling axis and contributes to BRAFV600E tumor growth (30, 31). In breast cancer, tumor cells, by increasing the number of MCT2 transporters at the plasmic membrane, are also able to take up β-OHB produced by adipocytes localized at the tumor site. This uptake favors the clonogenic potential of MCT2 positive tumor cells as well as their capacity to form a tumor mass *in vivo* (32).

Ammonia recycling by tumor cells is another example of how cancer cells benefit from the capture of metabolites released either into the TME by neighboring cells or in to the circulation by the liver. Ammonia produced during the conversion of glutamine to glutamate was, until recently, considered as waste. In a metabolic symbiosis context, Spinelli et al. recently demonstrated that the recycling of ammonia maximizes nitrogen utilization by glutamate dehydrogenase for glutamate synthesis from α-ketoglutarate. Glutamate then contributes to proline or aspartate synthesis. Importantly, the authors showed that this recycling accelerates proliferation of breast tumors (33). Hence, ammonia and KB recycling by tumors highlights the need to consider the interactions, in the pathophysiologic context of cancer, between tumors and the rest of the host organism. It is then crucial to integrate the metabolites exchange between the tumor and metabolic organs/tissues of the host in *ex vivo* studies of the metabolic reprogramming of tumors. Although the set up of *in vitro* and *in vivo* experimental models to consider such metabolic communication appears ambitious and complex, it emerges nowadays as a necessity to take tumor metabolism studies to a new level and improve the relevance of translational studies for effective metabolic therapies (34). In line with this, mathematic models of the tumor-organs metabolic interactions become essential tools in establishing experimental models.

## Metabolic Flexibility of Metastatic Cells

**Figure 2:**
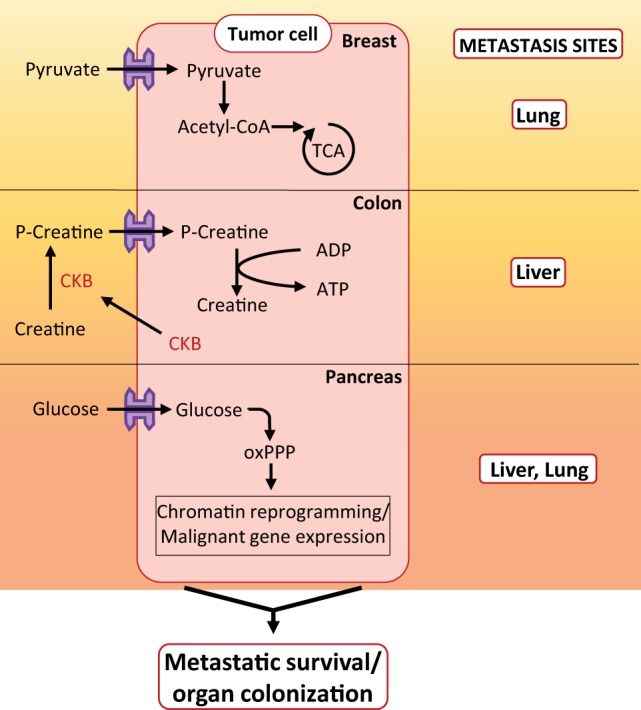
Metabolic phenomenon occurring in metastasis sites. Example of metabolic pathways activated in breast, colon, and pancreatic metastatic cells during colonization of lung, liver, and liver/lung, respectively. Abbreviations: oxPPP, oxidative branch of the pentose phosphate pathway; CKB, brain-type creatine kinase.

The metastasis sites’ microenvironment retains some similarity to the primary site for components such as collagens, hyaluronan, and smooth muscle actin-expressing cells (35), but also differs from the primary microenvironment in the level of their cellular/acellular components and available nutrients. Hence, tumor cells must develop a metabolic plasticity to satisfy their biomass and energetic demands required for their proliferation in both primary and metastasis sites. It is, therefore, important to highlight a few studies revealing the metabolic programs of metastatic cells according to their tissue of origin and/or to the metastasis site microenvironment. Christen and collaborators revealed that lung interstitial fluid has increased levels of pyruvate compared to blood plasma resulting in higher pyruvate levels in lung metastases compared to primary breast tumors. Consequently metastatic breast cancer cells take advantage of the pyruvate availability in their metastatic environment by increasing pyruvate carboxylase-dependent anapleurosis compared to primary tumor cells (36) (Figure 2). As a metabolic organ, the liver produces a plethora of metabolites usable by colonizing metastatic tumor cells, creatine being one of them. Creatine is phosphorylated by creatine kinase (CK), resulting in phosphocreatine, useful to shuttle high energetic phosphate when it enters into cells to produce ATP from ADP when energetic needs exceed ATP synthesis. When colon cancer cells metastasize in the liver, they must face the new environmental hypoxic conditions. Secretion of the brain-type CK (CKB) by colon metastatic cells into the extracellular space of the liver allows production of phosphocreatine from extracellular ATP and creatine. Phosphocreatine uptake by liver-disseminated metastatic cells supplies them with ATP to survive the hypoxic microenvironment (Figure 2). Consequently, inhibition of CKB in disseminated colon cancer cells impedes metastatic colonization of the liver. Inhibition of CKB and the creatine transporter in PDAC cells also limits their metastatic potential, highlighting that a common metabolic targeting of disseminated cells originating from different gastrointestinal organs is feasible to abolish metastatic progression (37). Interestingly, during the evolution of localized PDAC toward a metastatic disease, a widespread epigenetic reprogramming occurs. Moreover, metastasis at distant sites appears to be dependent on the oxidative branch of the PPP (oxPPP). Indeed, 6-phosphogluconate dehydrogenase, the enzyme responsible for nucleotide synthesis through the oxPPP, not only controls tumor growth at metastasis sites but also governs chromatin reprogramming as well as malignant gene expression. This illustrates that epigenetic events leading metastatic PDAC progression are dependent on the metabolic reprogramming of metastatic cells (38) (Figure 2). In metastasis sites, tumor cells also influence the CAFs’ metabolic activity. Indeed, breast cancer cells transfer miR-122 through EVs to resident fibroblasts in lung pre-metastatic niches. By reducing glucose consumption by fibroblasts, miR-122 increases glucose availability to tumor cells (39). Hence, metabolic remodeling of stromal cells in metastasis sites provides favorable “soil” for seeding and growth of cancer cells.

## Conclusion

Metabolic cooperation between the TME and cancer cells contributes to tumor growth, especially in nutrient- or oxygen-deprived microenvironments. As a consequence, targeting the metabolism of stromal cells impedes tumor progression to the same extent as targeting the tumor cells’ metabolic mediators. Therefore, when considering metabolic targeting of tumors as an anti-cancer therapy, targeting only the cancer cell autonomous metabolism would not be sufficient. Moreover, we highlight in this review that the nurturing microenvironment supplies tumor cells with macromolecules or metabolites and fuels metabolic pathways especially in stressful conditions. Fortunately, mechanisms used by tumor cells for the uptake of such environmental nutrients are being progressively uncovered. Development of therapeutic metabolic approaches must, therefore, take into account that the metabolic reprogramming of tumor cells is flexible and evolves along with microenvironmental changes. As such, a unique metabolic therapeutic window is not conceivable. This highlights the need to develop combined metabolic targeting to circumvent tumor metabolic plasticity and abolish tumor progression in the long term.

## Author Contributions

VG, FG, and SV conceived, organized, and wrote the manuscript.

## Conflict of Interest Statement

The authors declare that the research was conducted in the absence of any commercial or financial relationships that could be construed as a potential conflict of interest.
